# Remarkable stability of an instability-prone lentiviral vector plasmid in *Escherichia coli* Stbl3

**DOI:** 10.1007/s13205-012-0070-8

**Published:** 2012-07-27

**Authors:** Faisal A. Al-Allaf, Oleg E. Tolmachov, Lia Paola Zambetti, Viktoria Tchetchelnitski, Huseyin Mehmet

**Affiliations:** 1 Department of Medical Genetics, Faculty of Medicine, Umm Al-Qura University, Al-Abedia Campus, P. O. Box 715, Makkah, 21955 Saudi Arabia; 2 Division of Clinical Sciences, Faculty of Medicine, Institute of Reproductive and Developmental Biology, Imperial College London, London, W12 0NN UK; 3 Faculty of Medicine, National Heart and Lung Institute, Sir Alexander Fleming Building, Imperial College London, London, SW7 2AZ UK

**Keywords:** Plasmid instability, Lentiviral vector, *Escherichia coli*, Stbl3, Stbl2

## Abstract

Large-scale production of plasmid DNA to prepare therapeutic gene vectors or DNA-based vaccines requires a suitable bacterial host, which can stably maintain the plasmid DNA during industrial cultivation. Plasmid loss during bacterial cell divisions and structural changes in the plasmid DNA can dramatically reduce the yield of the desired recombinant plasmid DNA. While generating an HIV-based gene vector containing a bicistronic expression cassette 5′-Olig2cDNA-IRES-dsRed2-3′, we encountered plasmid DNA instability, which occurred in homologous recombination deficient *recA1 Escherichia coli* strain Stbl2 specifically during large-scale bacterial cultivation. Unexpectedly, the new recombinant plasmid was structurally changed or completely lost in 0.5 L liquid cultures but not in the preceding 5 mL cultures. Neither the employment of an array of alternative *recA1 E. coli* plasmid hosts, nor the lowering of the culture incubation temperature prevented the instability. However, after the introduction of this instability-prone plasmid into the *recA13E. coli* strain Stbl3, the transformed bacteria grew without being overrun by plasmid-free cells, reduction in the plasmid DNA yield or structural changes in plasmid DNA. Thus, *E. coli* strain Stbl3 conferred structural and maintenance stability to the otherwise instability-prone lentivirus-based recombinant plasmid, suggesting that this strain can be used for the faithful maintenance of similar stability-compromised plasmids in large-scale bacterial cultivations. In contrast to Stbl2, which is derived wholly from the wild type isolate *E. coli* K12, *E. coli* Stbl3 is a hybrid strain of mixed *E. coli* K12 and *E. coli* B parentage. Therefore, we speculate that genetic determinants for the benevolent properties of *E. coli* Stbl3 for safe plasmid propagation originate from its *E. coli* B ancestor.

## Introduction

Cloning a given DNA fragment within a plasmid vector is usually a straightforward procedure. However, from time to time experimenters encounter insert-vector combinations, which are difficult to assemble because of unexpected plasmid instability. In general, the nature of this plasmid instability is poorly understood. Plasmid instability can be influenced by a number of factors including the expression of toxic gene products, metabolic burden of plasmid maintenance, plasmid copy number, intense positive selection pressure for a particular plasmid segment (e.g. antibiotic resistance gene), the genotype of the host strain and the recombinogenic potential of sequences present in the plasmid (Corchero and Villaverde 1998). Indeed, on some occasions instability was observed to be associated with multiple repeats (Bichara et al. 2000, Kang and Cox 1996), AT-rich sequences or sequences prone to the formation of secondary structures (Razin et al. 2001). Both structural and maintenance (segregational) forms of plasmid instability occur, often simultaneously. Sometimes an extreme case of plasmid instability, where a nascent recombinant plasmid is fatally unstable, can be perceived as ‘unclonability’ of a specific DNA sequence. In practice, such fatal plasmid instability is difficult to discern from faults in a cloning technique. Faithful detection of ‘unclonability’ can be accomplished by coupling the analyses of bacterial transformants and the ligation mixture by duplex PCR (Al-Allaf et al. 2005). As a rule, the larger the plasmid and the more mosaic the origin of its sequences, the greater the likelihood that it will contain sequences that will be selected against by the host bacterium.

Overcoming plasmid instability and maintaining the plasmid DNA in an unchanged form can be a serious challenge. Optimal choice of both replication origin and plasmid partition machinery is known to be important for successful plasmid stabilization. A low cultivation temperature was reported to be beneficial for a higher stability, purportedly because of the reduced rate of bacterial growth (Liao 1991). Failing that, various *Escherichia coli* strains can be tested to find the host that is the most permissive for the faithful maintenance of a given recombinant plasmid. Several *E. coli* strains were reported to confer increased stability to problematic plasmids: *E. coli* Stbl2™ (Trinh et al. 1994) and Stbl4™ (Schmidt and Bloom 1999) from Invitrogen and *E. coli* SURE^®^ cells (Razin et al. 2001) from Stratagene.

Many laboratories, including our own, encountered difficulties while subcloning human sequences within the plasmid backbones of viral gene vectors. In an attempt to construct a lentiviral gene vector based on the human immunodeficiency virus 1 (HIV-1) for the transfer and expression of the human Olig2-cDNA sequence in human cells, we have been able to isolate a clone with the expected lentiviral vector backbone plasmid containing a bicistronic 5′-Olig2cDNA-IRES-dsRed2-3′ cassette in the desired orientation. However, in contrast to satisfactory maintenance and the structural stability of the plasmid during mini-scale plasmid propagation, plasmid DNA was structurally changed and/or completely lost in large-volume cultivation of the *E. coli* Stbl2 plasmid host. We have evaluated the instability systematically and showed that by changing the host strain only to *E. coli* Stbl3, it is possible to maintain the instability-prone plasmid at a high copy number in a large-scale culture.

## Materials and methods

### Bacterial strains and plasmids

The *E. coli* strain Stbl2 is a derivative of *E. coli* JM109 and has the following genotype: F^−^ endA1 glnV44 thi1 recA1 gyrA96 relA1 Δ(lac-proAB) mcrA Δ(mcrBC-hsdRMS-mrr) λ^−^. The *E. coli* strain Stbl3 is derived from *E. coli* HB101 and has the following genotype: F^−^*mcr*B *mrr hsd*S20 (rB^−^, mB^−^) *rec*A13 *sup*E44 *ara*14 *gal*K2 *lac*Y1 *pro*A2 *rps*L20 (Str^R^) *xyl*5 λ^−^*leu mtl*1. *E. coli* DH5α is F^−^ ϕ80d*lacZ*∆M15 ∆(*lacZ*YA-*arg*F)U*169 end*A1 *rec*A1 *hsd*R17 (r_K_^−^ m_K_^+^) *deo*R *thi*1 *pho*A *sup*E44 λ^−^*gyr*A96 *rel*A1. The *E. coli* DH10B is F^−^*mcr*A ∆(*mrr*-*hsd*RMS-*mcr*BC) ϕ80d*lacZ*∆M15 ∆*lac*X74 *end*A1 *rec*A1 *deo*R ∆(*ara, leu*)7,697 *ara*D139 *gal*U *gal*K *nup*G *rps*L λ^−^. *E. coli* Top10 is F^−^*mcr*A Δ(*mrr*-*hsd*RMS-*mcr*BC) ϕ80*lac*ZΔM15 Δ*lac*X74 *rec*A1 *ara*D139 Δ(*ara*-*leu*)7,697 *gal*U *gal*K *rps*L (Str^R^) *end*A1 *nup*G. All the bacterial strains were purchased from Invitrogen. Plasmids used were pIRESdsRed2 (Clontech), pRRE.SIN.cPPT.PGK.eGFP.WPRE (kindly provided by Prof Luigi Naldini, Italy), pBluscriptR-Olig2 (kindly provided by Dr. John Alberta, Dana–Farber Cancer Institute, USA). All DNA manipulations were performed as described previously (Sambrook et al. 1989).

### PCR amplification

PCR amplification was carried out in 30 μl of a reaction mixture containing 100 ng template DNA, 1–2 μl ligation mixture or a loopful of a single bacterial colony, 23 μl dH_2_O, 0.5 μl of (5 U/μl) Accuzyme DNA polymerase (Bioline), 3 μl of 10× PCR buffer (Bioline), 1 μl of 10 mM dNTPs (Invitrogen), 1 μl of 20 pmol forward primer HIV-PGK-F1 (5′-GACCGAATCACCGACCTCTC-3′) or forward primer HIV-Olig2-F2 (5′-GGACAAGCTAGGAGGCAGTG-3′), 1 μl of 20 pmol backward primer HIV-Olig2-B1 (5′-CACCAGTCGCTTCATCTCCT-3′) and 1 μl of 50 mM MgCl_2_. The reactions were run on the Primus HT thermocycler (MWGAG Biotech) with the following program: 40 cycles of PCR were carried out, each cycle included 1 min at 94 °C, 1 min at 58 °C, and 1 min at 72 °C followed by a final extension at 72 °C for 10 min. When bacterial colonies were used as a source of template DNA, the reaction mixtures were pre-incubated at 94 °C for 3 min to lyse the cells. The expected 320 and 653 bp PCR amplicons were then size-fractionated by electrophoresis in a 1.5 % (w/v) agarose gel alongside 100 bp ladder (Promega) as a molecular marker. All the employed primers were designed using the software at http://frodo.wi.mit.edu/cgi-bin/primer3/primer3_www.cgi and purchased from Thermo Electron GmbH, Ulm, Germany.

### Growth conditions

The bacterial strains were grown in SOC and LB broth (Invitrogen) or on LB agar medium supplemented with 100 μg/ml of ampicillin (*Ap*) or 50 μg/ml of kanamycin as required. Bacterial cultures of 5 ml or 500 ml were inoculated with a single colony loopful or starter culture (1 % v/v), respectively, and incubated in an orbital shaker (200 rpm) at 30 or 37 °C. Samples were taken from the bacterial cultures and the cells were harvested by centrifugation for 15 min at 11,500*g*. Subsequent plasmid DNA preparations were performed using the QIAMini- or QIAMaxi-prep kits according to the manufacturer’s protocols (Qiagen). For long-term storage, a saturated bacterial culture was frozen at −80 °C in the presence of 50 % (v/v) glycerol.

### Analysis of plasmid stability

Typically, a single colony was picked from a selective nutrient agar plate, inoculated into 5 ml of LB broth and incubated at 37 °C with shaking at 200 rpm for 18 h. Aliquots from this initial stationary phase culture were then used for plasmid DNA mini-preparation, glycerol stock preparation or plasmid instability analysis. Due to the plasmid instability observed in pilot experiments after transformation of the Stbl2 strain, it was decided to use the initial stationary phase culture for the starting inoculum instead of a derivative starter culture. Thus, in the long-term plasmid stability experiment, 100 μl inoculates of the glycerol stock of the original plasmid-harbouring culture were added to the 50 ml Falcon tubes containing 9.9 ml of LB medium supplemented with ampicillin (*Ap)* and all the cultures were grown at 37 °C in a shaking incubator (200 rpm) for 30 h. Alternatively, where indicated, 5 ml inoculates were used to seed 500 ml cultures grown in 2 L Erlenmeyer flasks. Samples were collected at time points and analyzed by measuring the OD_600_ using a spectrophotometer (Aquarius, Cecil Elegant Technology). In addition, culture samples from each time point were diluted (10^−2^, 10^−4^, 10^–6^, 10^−8^) and plated onto LB agar plates, the resultant colonies were then replicated on LB agar plates with and without antibiotic selection. Fractions of *Ap*-resistant clones were used to produce an estimate of segregational plasmid stability. Next, 1.5 ml samples of the cultures were collected in Eppendorf tubes and the plasmid DNA was extracted with QIAprep Spin Miniprep Kit Protocol (Qiagen). Then the purified DNA from the colonies corresponding to all the time points were analyzed via digestion with *Bgl*II restriction endonuclease (Invitrogen).

### Molecular cloning

The declared aim was to generate a lentiviral HIV-based vector for the transfer and simultaneous expression of both the human Olig2-cDNA and dsRed fluorescent marker gene. We first constructed a 5′-Olig2cDNA-IRES-dsRed-3′ bisictonic cassette by subcloning the human Olig2-cDNA into the pIRES2-dsRed2 plasmid backbone (Clontech). The pBluscriptR-Olig2 plasmid was digested with *Sac*I and then ligated to *Sac*I-digested and alkaline phosphatase-treated pIRES2-DsRed2 vector. The ligation mixture was then used to transform chemically competent cells of the *E. coli* strain Stbl2 (Invitrogen). Plasmid clones with the desired orientation of the insert were found and confirmed by restriction analysis. The 5′-Olig2-IRES-DsRed-3′ cassette was then excised from the pOlig2cDNA-IRES2-DsRed2 expression plasmid using the *Nhe*I and the *Mfe*I restriction enzymes (Invitrogen), blunt-ended with Klenow large fragment polymerase I (Roche) and then ligated to *Bam*HI/*Sal*I digested, blunt-ended and treated by alkaline phosphatase (Promega) lentiviral backbone plasmid pRRL.SIN.cPPT.PGK/GFP.WPRE. The ligation was performed using T4 ligase (Invitrogen). After 20 h of incubation at 4 °C, the ligation mixture was examined for its usefulness for bacterial transformation by PCR detection of a site-specific fragment insertion using a duplex primer pairs. The positive ligation sample-PCR was then used to transform DH5α, Top10, DH10B, Stbl2 and Stbl3 chemically competent cells (Invitrogen) according to the manufacturer’s instructions. Aliquots of the transformation suspensions were plated on LB agar medium supplemented with *Ap* (100 μg/ml, Sigma). Following overnight incubation at 30 or 37 °C, single colonies were screened by duplex PCR using insert-insert and insert-vector primer pairs for the selection of clones harbouring the plasmid with the desired orientation of the 5′-Olig2-IRES-DsRed-3′ insert. The DNA of the PCR-positive plasmid clone was sequenced from the primer HIV-PGK-F1 to confirm the insert orientation. The confirmation of the functional expression of olig2 protein was described previously (Kennea et al. 2009).

## Results

### Screening for a suitable *E. coli* strain to establish replication of the lentiviral vector plasmid containing Olig2 cDNA

This work was done in the context of the generation of an HIV-based lentiviral vector construct with the human Olig2-cDNA and the dsRed fluorescent marker gene (Soneoka et al. 1995). The bicistronic cassette containing the 5′-Olig2-IRES-dsRed2-3′ has been successfully joined to the high copy number HIV-based lentiviral plasmid pRRL.SIN.cPPT.PGK-eGFP-WPRE by ligation in vitro. Proof of successful ligation was obtained by the duplex PCR amplification method with insert-insert and insert-vector primer pairs (Al-Allaf et al. 2005). However, repeated attempts of transformation of competent cells of *E. coli* strains DH5α, Top10 and DH10B with the obtained ligation mixture failed to produce transformant colonies with incubation of Petri dishes both at the standard temperature of 37 °C and at a lowered temperature of 30 °C.

Similar results indicating DNA sequence ‘unclonability’ were observed by some researchers, who managed to minimize the ‘unclonability’ problem by using an alternative strain of the host bacteria (Al-Allaf et al. 2005; Razin et al. 2001; Trinh et al. 1994). Therefore, the *E. coli* Stbl2 strain, which was previously reported to improve the cloning efficiency of an insert containing a DNA sequence repeat (Trinh et al. 1994) and an unstable viral plasmid (Al-Allaf et al. 2005; Trinh et al. 1994) was transformed using the same ligation mixture. Only four colonies were obtained and were then used to inoculate individual overnight cultures in 5 ml of LB broth containing *Ap* in order to perform plasmid DNA mini-preparations and subsequent analyses.

### Restriction analysis of the recombinant plasmid hosted by *E. coli* Stbl2 and *E. coli* Stbl3

**Fig. 1:**
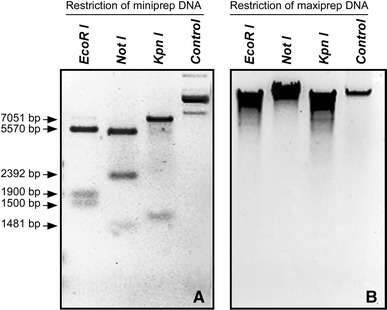
Restriction analysis of plasmid DNA extracted from the *E. coli* Stbl2 strain, which was transformed with the pRRL.SIN.cPPT.PGK/Olig2-IRES-DsRed-WPRE plasmid. **a** An agarose gel analysis of restriction digestions of the plasmid DNA minipreparations confirming the expected restriction patterns with *Eco*RI, *Not*I and *Kpn*I and the expected total plasmid size of 9.1 kb. **b** An agarose gel analysis of the restriction digestions of the plasmid DNA maxi-preparation with *Eco*RI, *Not*I and *Kpn*I showing structural changes in the DNA, which indicate plasmid DNA instability. Control–undigested plasmid DNA

Restriction analysis of the four plasmid DNA mini-preparations with the enzymes *Eco*RI, *Not*I and *Kpn*I showed the expected restriction patterns and the expected 9.1 kb total size of the new recombinant plasmid pRRL.SIN.cPPT.PGK/Olig2-IRES-DsRed-WPRE with the desired orientation of the 5′-Olig2cDNA-IRES-dsRed-3′ insert in one of the four clones (Fig. 1a). No plasmid DNA or a very low amount of incorrectly sized plasmid DNA was detected in the other three clones (data not shown). As the DNA yield after mini-preparation of the plasmid DNA does not provide enough DNA for a large-scale production of the desired infectious viral particles, we scaled up the Stbl2 bacterial culture from 5 ml to 500 ml. After maxi-preparation of plasmid DNA, restriction digestions with *Eco*RI*, Not*I, and *Kpn*I showed structural changes in the plasmid DNA with the unexpected presence of high molecular weight DNA (Fig. 1b) indicating plasmid DNA instability.

**Fig. 2:**
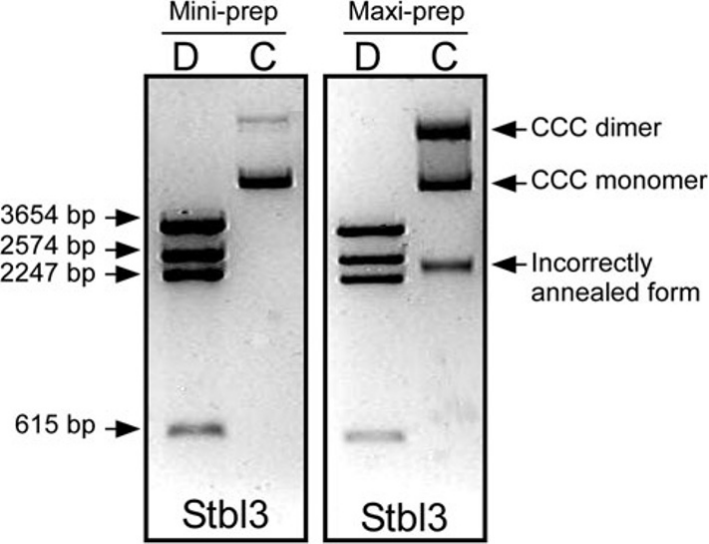
Restriction analysis of the pRRL.SIN.cPPT.PGK/Olig2-IRES-DsRed-WPRE plasmid DNA propagated in *E. coli* Stbl3 cells. An agarose gel analysis of *Bgl*II-digested plasmid DNA extracted as mini-preparations and a maxi-preparation after propagation in Stbl3 cells showed the expected *Bgl*II restriction pattern and the expected total plasmid size indicating faithful plasmid maintenance in Stbl3 cells. *d* Digested plasmid DNA, *c* Control undigested plasmid DNA, *CCC* covalently closed circular DNA

Then we used an aliquot of the pRRL.SIN.cPPT.PGK/Olig2-IRES-DsRed-WPRE plasmid DNA obtained after DNA mini-preparation (shown in Fig. 1a) for the transformation of the *E. coli* Stbl3. Unlike examination of the Stbl2-extracted DNA, *Bgl*II restriction analysis of the plasmid DNA isolated as both mini- and maxi-preparations from the Stbl3-host showed the correct 9.1 kb plasmid size with no apparent structural changes in the plasmid DNA being detected in all the studied clones (Fig. 2). Thus, structural and maintenance stability of the plasmid was observed when the Stbl3 strain, but not Stbl2 strain, was used as a plasmid host.

### Evaluation of the recombinant plasmid stability in Stbl2 and Stbl3

We then evaluated the plasmid stability in both the Stbl2 and the Stbl3 *E. coli* strains by estimating fractions of plasmid-harbouring cells during bacterial cultivation. An aliquot of pRRL.SIN.cPPT.PGK/Olig2-IRES-DsRed-WPRE plasmid DNA obtained after the original Stbl2 DNA mini-preparation was used again to transform both the Stbl2 and the Stbl3 bacterial strains. Colonies were selected randomly and individually inoculated into overnight cultures in LB medium for plasmid DNA mini-preparation. Restriction analysis of DNA mini-preparations from the clones of each strain confirmed the production of the plasmid with the expected restriction pattern and the expected total size of 9.1 kb, corroborating previous observations.

**Fig. 3:**
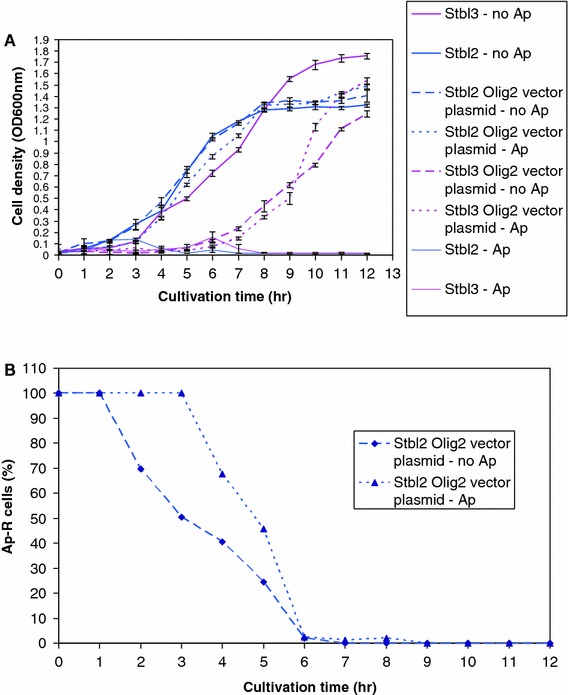
Monitoring of bacterial growth and plasmid maintenance analysis for bacterial cultures harbouring the pRRL.SIN.cPPT.PGK/Olig2-IRES-DsRed.WPRE plasmid. The data for the Stbl3-based strains are presented as *pink lines*, the data for the Stbl2-based strains are presented as *blue lines*. **a** Cell density monitoring in the growing cultures of the Stbl2 and the Stbl3 strains, naïve or transformed with the pRRL.SIN.cPPT.PGK/Olig2-IRES-DsRed.WPRE plasmid. Cells were grown in 500 ml of LB media in the presence and absence of *Ap* at 37 °C in 2 L Erlenmeyer flasks, with inoculation from a 5 mL overnight culture, with aeration by shaking. Cell density was measured as optical density (OD_600_). The data are presented as averages from 5 biological repeats with the error bars corresponding to standard deviations. **b** The projected percentage of the plasmid-harbouring *Ap*-resistant cells, which was estimated after seeding dilutions of the samples obtained from the growing cultures onto non-selective LB agar and replicating 155 resultant colonies onto selective (supplemented with *Ap*) and non-selective LB agar plates. Data are presented graphically for the plasmid-harbouring Stbl2 strain only. In each culture growth time point all 155 clones (100 %) of the plasmid-harbouring Stbl3 strain were resistant to *Ap*, while none of the tested clones (0 %) of the non-transformed Stbl2 and Stbl3 strains were resistant to *Ap*

We then used aliquots of the obtained Stbl2-based and Stbl3-based overnight cultures to inoculate flasks with 500 ml of medium to grow the new cultures in the presence or absence of *Ap* for both strains. We collected the culture samples each hour in a 12-h time interval for OD_600_ measurements and the plasmid stability assays (Fig. 3). 4 h after bacterial inoculation, both transformed strains were in transition to the exponential phase. However, while the Stbl2-based plasmid-harbouring strain grew fast and in general congruence to the non-transformed Stbl2, the Stbl3-based plasmid-harbouring strain grew weakly and markedly slower than the non-transformed Stbl3. The transformed Stbl3 strain was likely to grow poorly because it was faithfully maintaining the recombinant plasmid, which inhibited bacterial growth, while the transformed Stbl2 strain rapidly lost the plasmid and, therefore, was not restrained by it. No dramatic effect of *Ap* on the culture growth curves was observed for both of the transformed strains; this was likely to be due to rapid destruction of *Ap* by β-lactamase secreted by the plasmid-harbouring cells.

Plasmid maintenance stability was assessed by plating aliquots from each time point on non-selective plates with subsequent replication of the colonies on *Ap*-free and *Ap*-supplemented nutrient agar media. After overnight incubation at 37 °C, the fractions of *Ap*-resistant clones were determined (Fig. 3b). Unsurprisingly, the estimated percentage of plasmid-carrying Stbl2 cells was shown to decline after less than 2 h when cultured in the presence of *Ap* selection and after 3 h when cultured in the absence of *Ap* selection. However, the percentage of plasmid-carrying Stbl3 cells was estimated to be 100 % throughout the entire 12 h interval, indicating the stability and maintenance of the plasmid DNA in the Stbl3 strain. No effect of *Ap* selection on the plasmid stability in Stbl3 strain could be observed.

We then examined the long-term segregational stability of the plasmid in the transformed Stbl3 strain. Thus, the timeframe for the plasmid stability studies was extended and the results showed that the pRRL.SIN.cPPT.PGK/Olig2-IRES-DsRed.WPRE plasmid was efficiently maintained in the Stbl3 strain with growth in selective and non-selective media and daily passaging over 5 days, while the re-transformed Stbl2, Top10, DH10B and DH5α strains did not retain the plasmid after the initial 2–3 h of growth (data not shown).

**Fig. 4:**
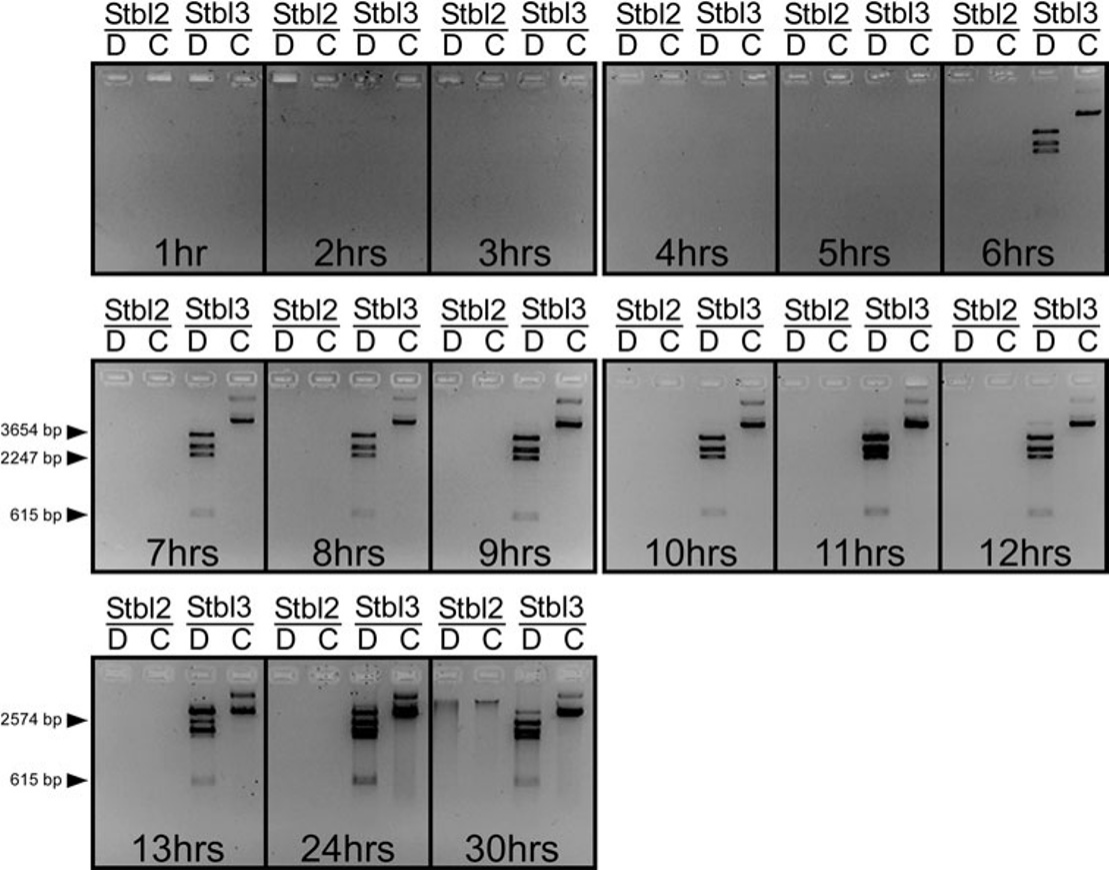
Identification of plasmid DNA instability by *Bgl*II restriction analysis of plasmid DNA extracted from bacterial culture aliquots collected at the individual time points during large-scale bacterial growth. No plasmid DNA was detected by an agarose gel analysis in the growing *E. coli* Stbl2 bacteria transformed with pRRL.SIN.cPPT.PGK/Olig2-IRES-DsRed.WPRE plasmid at any time point with the only exception of the 30-h sample, where the extracted DNA was structurally changed. In contrast, an agarose gel analysis of *Bgl*II digestions of plasmid DNA isolated from the large-scale culture of the transformed *E. coli* Stbl3 strain showed four *Bgl*II-fragments with the expected sizes of 3,654, 2,574, 2,247, 615 bp starting from the 6-h time point onwards, suggesting the plasmid’s stability in the Stbl3 strain. *d* Digested plasmid DNA, *c* Control undigested plasmid DNA

To test whether the instability of the pRRL.SIN.cPPT.PGK-Olig2-IRES-DsRed-WPRE plasmid was due to the loss of the whole plasmid or to structural changes in the plasmid, we extracted plasmid DNA from each time point aliquots. Figure 4 shows restriction analysis of extracted DNA using *Bgl*II endonuclease. No DNA from Stbl2 transformed cells was detected in any time point, with the exception of the 30 h time point where the extracted DNA was structurally different compared to the initial mini-preparation shown in Fig. 1a. On the contrary, restriction digestions of DNA retrieved from the Stbl3 strain starting from 6-h time point onwards showed the expected four *Bgl*II-fragments with the sizes of 3,654, 2,574, 2,247, 615 bp, thereby indicating the structural stability of the clones.

**Fig. 5:**
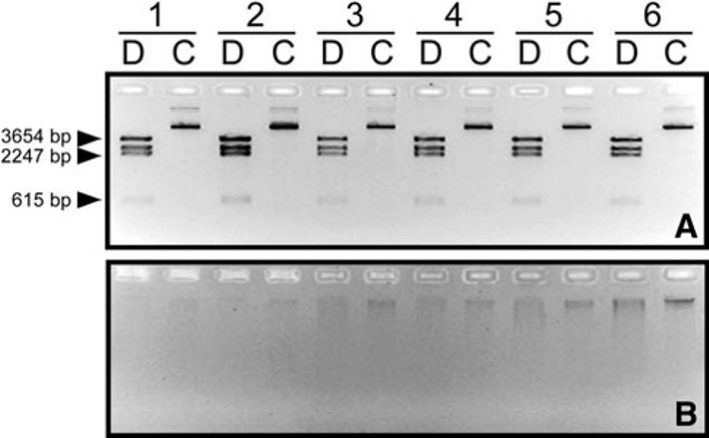
Restriction analysis of the pRRL.SIN.cPPT.PGK/Olig2-IRES-DsRed-WPRE plasmid DNA propagated in Stbl2 and in Stbl3 cells. **a** An agarose gel analysis of *Bgl*II restriction digestions showing the expected fragmentation pattern (3,654, 2,574, 2,247, 615 bp fragments) for plasmid DNA extracted from Stbl3 transformants. **b** An agarose gel analysis of *Bgl*II restriction digestions of plasmid DNA extracted from *Ap*-resistant transformants of the Stbl2 strain, indicating that the plasmid is either completely lost or structurally changed. *d* digested plasmid DNA, *c* control undigested plasmid DNA

To provide further evidence of the structural stability of the pRRL.SIN.cPPT.PGK-Olig2-IRES-DsRed-WPRE plasmid in the Stbl3 host, we plated the cells from each time point and performed random colony screening by duplex colony PCR using insert-insert and vector-insert primer pairs. The screening showed the generation of expected 310 and 656 bp amplicons in 33 out 34 PCR (97 %) indicating 5′-Olig2cDNA-IRES-dsRed2-3′ maintenance in these 33 bacterial clones. The remaining one clone (3 %) was positive for one amplicon only (data not shown). Ten randomly selected duplex PCR-positive colonies were then individually incubated in LB growth medium supplemented with *Ap* for maxi-preparation of plasmid DNA. Restriction analysis using *Bgl*II showed the expected pattern of digestion (3,654, 2,574, 2,247, 615 bp fragments) proving that pRRL.SIN.cPPT.PGK-Olig2-IRES-DsRed-WPRE DNA was structurally stable and well maintained in the *E. coli* Stbl3 strain (Fig. 5a). In comparison, all 10 screened colonies, grown from individual bacteria from the 500 ml liquid culture of the Stbl2 transformant, were PCR-negative suggesting the loss of the entire plasmid (Fig. 5b).

The presented results prove the capability of Stbl3 cells to stably maintain an erratic HIV-based plasmid pRRL.SIN.cPPT.PGK/Olig2-IRES-DsRed-WPRE and suggest that the problems encountered in the initial establishment of this plasmid in bacterial cells and its loss in large-scale bacterial culture were due to poor suitability of the initially employed Stbl2 strain to support faithful replication of this plasmid.

## Discussion

It is well known that plasmid instability often presents difficulties in laboratory manipulation of unstable genetic sequences (Al-Allaf et al. 2005; Boyd et al. 1999; Razin et al. 2001; Yudinkova et al. 2001). Plasmids constructed for gene therapy and/or genetic vaccinations are made from DNA sequences, which might be taken from bacteria, viruses, animals and humans. The nascent plasmid can inhibit the growth of the bacterial host or even kill it, can undergo a structural rearrangement or can be rapidly lost during cell divisions. These signs of plasmid instability can manifest themselves at the stage of the initial transformant colony formation or during subsequent expansion of the bacterial culture and sometimes could be fatal for the newly created plasmid chimeras. It is noteworthy that the majority of reported instability occurrences are observed in attempts to clone eukaryotic DNA in constructs propagated in prokaryotic host (Al-Allaf et al. 2005; Boyd et al. 1999; Razin et al. 2001; Shirsat et al. 1992; Wyman et al. 1985). Fragments with toxic genes (Vidal et al. 1991), a long inverted DNA repeat separated by a short intervening segment (Prats et al. 1985), multiple direct repeats (Bichara et al. 2000; Kang and Cox 1996), AT-rich sequences and sequences capable of forming cruciform-like structures (Razin et al. 2001) or other non-B DNA structures (Catasti et al. 1996; Murchie and Lilley 1992; Seigneurin et al. 1988; Simonsson et al. 1998) are known to contribute to plasmid instability.

The reason for the instability of the newly generated plasmid pRRL.SIN.cPPT.PGK/Olig2-IRES-DsRed.WPRE in a large-volume culture of *E. coli* Stbl2 is not known. However, as this plasmid contains the inverted DNA sequence of the HIV-1 long terminal repeat (LTR) flanking the 5′-Olig2cDNA-IRES-DsRed-3′ cassette and also sequences capable of forming G-quadruplexes (Lyonnais et al. 2002) that are particularly prone to rearrangement, it can be assumed that these sequences might provoke the observed instability. However, these compromising factors are not sufficient to cause instability by themselves as the original pRRL.SIN.cPPT.PGK.eGFP.WPRE plasmid was successfully propagated and maintained in DH5α, Top10, and DH10B and Stbl2 cells. Unexpectedly, the stable pRRL.SIN.cPPT.PGK.eGFP.WPRE viral plasmid became instability-prone after insertion of the 5′-Olig2cDNA-IRES-DsRed-3′ cassette and subsequent bacterial growth in a high volume bacterial culture of *E. coli* Stbl2 host.

Another possible explanation for our data can be provided by the detrimental effects of the 5′-Olig2cDNA-IRES-dsRed2-3′ insert to the bacterial host cells, which are caused by the presence of a cryptic bacterial promoter driving the expression of a harmful protein (Bieth et al. 1997). For example, Yike and colleagues (Yike et al. 1996) tried to clone large peptide fragments of the cystic fibrosis transmembrane conductance regulator (CFTR) and discovered that the fragments of the protein containing its R domain were particularly toxic to the bacterial strain used for cloning (*E. coli* BL21/DE3). These authors proposed that the CFTR peptides were interacting and interfering with bacterial permeases (which are known to contain homologies to CFTR) resulting in bacterial toxicity. Indeed, the poor growth of the plasmid-transformed Stbl3 strain might be due to the bacterial growth-limiting effect of the 5′-Olig2cDNA-IRES-dsRed2-3′ insert within the context of the pRRL.SIN.cPPT.PGK vector.

*Ap* resistance is a common plasmid selection marker in commercially available lentiviral backbone plasmids. This choice, at least in part, is due to the need to limit the spectrum of antibiotic resistance genes in bacterial strains used for a large-scale production of plasmid DNA and, thus, to prevent the escape of multiple antibiotic resistance genes into the environment, which can contribute to the spread of multi-drug resistant pathogens. A downside of the *Ap* resistance marker is, however, the fact that the *Ap* resistance enzyme, β-lactamase, is secreted by plasmid-harbouring bacteria with ensuing fast destruction of *Ap* in liquid cultures. *Ap*’s mode of action relies on stalling the growth of sensitive bacteria by interference with their cell wall rather than on causing immediate bacterial death. Secretion of β-lactamase and the non-lethal action of *Ap* provide a fertile ground for the accumulation of plasmidless *Ap*-sensitive bacteria in liquid bacterial cultures relying on the selection of plasmid-harbouring cells with *Ap*. This phenomenon provides the most likely explanation for our observation of apparent segregational instability of pRRL.SIN.cPPT.PGK/Olig2-IRES-DsRed.WPRE plasmid in the Stbl2-host grown in 0.5 L liquid cultures supplemented with *Ap* as a selection agent. The suggested alternatives are either the employment of secretion-deficient, cytosolic, versions of β-lactamase (Tolmachov et al. 2011) or the replacement of *Ap* with its more stable analogue carbenicillin.

Plasmid markers conferring resistance to an anti-microbial substance with a lethal mode of action, e.g. the kanamycin resistance gene from transposon Tn5, can provide more strict bacterial selection than the *Ap* resistance gene. However, too stringent selection against plasmidless cells in the mixed population of plasmid containing and plasmidless cells might provoke structural plasmid instability as spontaneous plasmid deletion mutants could replicate faster, provide a higher copy number of the resistance gene and thus greater resistance to the anti-microbial substance with the ensuing outcompeting of the original plasmid by its deletion mutants. Perhaps the resultant structural instability is an additional reason why relatively ‘mild’ *Ap* selection is the universally favoured selection for the lentiviral vector backbone plasmids.

The idea of switching over to an alternative DNA cloning host to achieve increased plasmid stability was previously implemented with great advantage, e.g. when yeast was employed as a host organism to enable the cloning of the entire human mitochondrial genome (Bigger et al. 2011). It is also well known that the ability of bacterial cells to maintain unstable DNA is a strain-specific property and can be regulated via the introduction of specific mutations into the host genome. Thus, *E. coli* strains carrying *recA* mutations are widely accepted as reliable plasmid hosts (Zhao et al. 2007). Indeed, RecA protein is a homology-dependent DNA-strand-transferase, which can be a constant source of rare recombination errors leading to plasmid DNA rearrangements. Many standard *E. coli* cloning hosts, including *E. coli* Stbl2 contain *recA1* allele of the *recA* mutation (Trinh et al. 1994). In contrast, *E. coli* Stbl3 contains *recA13* allele of the *recA* mutation. It is tempting to link the observed strain-specific differences in the stability of the obtained chimerical plasmid to the inter-strain difference in *recA* alleles. However, both *recA1* and *recA13* mutations of the *recA* gene are known to result in severely recombination defective forms of the RecA protein (Kowalczykowski 1991). Thus, although all the other bacterial strains, which we unsuccessfully tried to use as the cloning hosts for this plasmid (DH5α, Top10 and DH10B), were also *recA1*, it is not likely that the difference in the *recA* alleles was the pivotal strain distinction, which was responsible for the observed failure of Stbl2 to maintain the pRRL.SIN.cPPT.PGK/Olig2-IRES-DsRed.WPRE viral plasmid in large-scale cultivations. Another common feature of *E. coli* Stbl2, DH5α, Top10 and DH10B is that all these strains are derivatives of the wild type isolate *E. coli* K12. In contrast, *E. coli* Stbl3 is a derivative of *E. coli* HB101 and is, therefore, a cross between the wild type *E. coli* isolates K12 and B. Thus, we favour the possibility that the crucial genetic determinants of the pRRL.SIN.cPPT.PGK/Olig2-IRES-DsRed.WPRE stability have originated from *E. coli* B.

The stability of the pRRL.SIN.cPPT.PGK/Olig2-IRES-DsRed.WPRE plasmid in *E. coli* Stbl3 and its instability in *E. coli* Stbl2 are indeed remarkable, as the Stbl2 strain has, on many occasions, been successfully used to clone unstable DNA. Thus, Strader and Howell (Strader and Howell 1997) used this strain to propagate a tandem array of four dihydropholate reductase (DHFR) genes, whereas other *E. coli* strains in that study (SURE, Stratagene and JM107, Fermentas) gave high frequencies of undesired recombination and reduction of the insert size. In 1995, Singh and Singh used Stbl2 to propagate a 2,333 bp-fragment of the potato virus Y (PVY), which was unstable in another common *E. coli* DNA cloning strain (DH5α) (Singh and Singh 1995). Similarly, Senechal et al. found that Stbl2 was the only bacterial host strain suitable for the cloning of the destabilizing DNA fragment encoding the full-length LGI4 gene, a member of a family of genes involved in epilepsy (Senechal et al. 2005).

Some eukaryotic sequences are known to contain GC-rich DNA regions called CpG-islands. Analysis of the pRRL.SIN.cPPT.PGK/Olig2-IRES-DsRed.WPRE sequence with GrailEXP software (http://compbio.ornl.gov/grailexp/) revealed that this plasmid contained 6 CpG slands. Three hydrogen bonds between complementary C and G nucleotides ensure a stronger binding than two hydrogen bonds between complementary A and T nucleotides. Consequently, Watson–Crick duplex in GC-rich DNA segments is known to be exceptionally stable. Replication of the pRRL.SIN.cPPT.PGK/Olig2-IRES-DsRed.WPRE is driven by the ColE1-type origin of replication; such replication necessarily involves the unwinding of the complementary DNA strands. Thus, it is reasonable to propose that, as the plasmid replication progresses, it becomes slower in the GC-rich DNA segments of pRRL.SIN.cPPT.PGK/Olig2-IRES-DsRed.WPRE and that this replication deceleration can result both in the general slow-down of the plasmid propagation with ensuing maintenance plasmid instability and the counter-selection against the GC-rich DNA segments with ensuing structural plasmid instability.

In the present study, we showed that changing the host *E. coli* strain alone enables the production of stable bacterial clones harbouring the desired pRRL.SIN.cPPT.PGK/Olig2-IRES-DsRed.WPRE plasmid. The results presented here are the first demonstration that *E. coli* Stbl3 is superior to *E. coli* Stbl2 in the maintenance of an instability-prone HIV-based plasmid. Based on the presented data, it can be suggested that *E. coli* Stbl3 might be a host of choice for propagating unstable DNA of lentiviral backbone plasmids. The presented plasmid stabilization scenario underscores a broadly applicable bacterial strain screening strategy for the reliable establishment and the faithful propagation of stability-compromised recombinant plasmids.
